# Ubiquitin-dependent and -independent functions of OTULIN in cell fate control and beyond

**DOI:** 10.1038/s41418-020-00675-x

**Published:** 2020-12-07

**Authors:** Nadine Weinelt, Sjoerd J. L. van Wijk

**Affiliations:** Institute for Experimental Cancer Research in Pediatrics, Goethe-University, Komturstrasse 3a, 60528 Frankfurt am Main, Germany

**Keywords:** Biochemistry, Cell biology

## Abstract

Ubiquitination, and its control by deubiquitinating enzymes (DUBs), mediates protein stability, function, signaling and cell fate. The ovarian tumor (OTU) family DUB OTULIN (FAM105B) exclusively cleaves linear (Met1-linked) poly-ubiquitin chains and plays important roles in auto-immunity, inflammation and infection. OTULIN regulates Met1-linked ubiquitination downstream of tumor necrosis factor receptor 1 (TNFR1), toll-like receptor (TLR) and nucleotide-binding and oligomerization domain-containing protein 2 (NOD2) receptor activation and interacts with the Met1 ubiquitin-specific linear ubiquitin chain assembly complex (LUBAC) E3 ligase. However, despite extensive research efforts, the receptor and cytosolic roles of OTULIN and the distributions of multiple Met1 ubiquitin-associated E3-DUB complexes in the regulation of cell fate still remain controversial and unclear. Apart from that, novel ubiquitin-independent OTULIN functions have emerged highlighting an even more complex role of OTULIN in cellular homeostasis. For example, OTULIN interferes with endosome-to-plasma membrane trafficking and the OTULIN-related pseudo-DUB OTULINL (FAM105A) resides at the endoplasmic reticulum (ER). Here, we discuss how OTULIN contributes to cell fate control and highlight novel ubiquitin-dependent and -independent functions.

## Graphical abstract

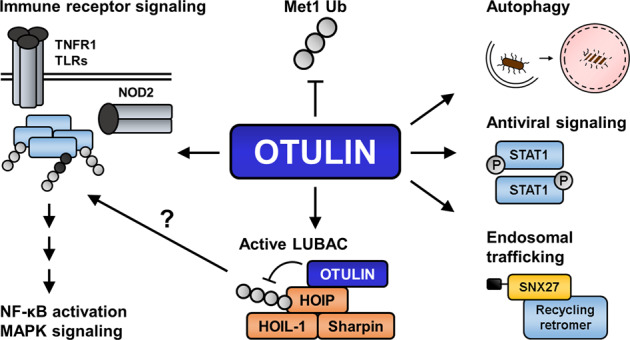

## Facts

- OTULIN is an OTU DUB that exclusively hydrolyzes Met1-linked poly-ubiquitination.
- OTULIN interacts with the LUBAC component HOIP and controls Met1 ubiquitination levels, NF-κB activation and cell fate in cell- and context-dependent cellular settings.
- OTULIN is involved in regulating interferon (IFN)/antiviral signaling and autophagic clearance of intracellular bacteria, as well as proteasomal degradation of cellular aggregates.
- Novel ubiquitin-independent functions of OTULIN and OTULINL have started to emerge at specific subcellular organelles.

## Open questions

- Is OTULIN statically interacting with LUBAC or in dynamic equilibrium with CYLD or other factors, and how relates this to DUB-E3 functions, target specificity and subcellular control of Met1 ubiquitination?
- Does OTULIN have additional cytosolic functions and do these rely on the LUBAC interaction or Met1 ubiquitin hydrolysis?
- How does OTULIN control autophagy and mTOR signaling?
- By what means is the activity of OTULIN regulated by external factors, like post-translational modifications or protein interactions, and how do these affect OTULIN function?
- What is the cellular role of OTULINL and how does it relate to OTULIN?

## Introduction

The control of ubiquitination, i.e., the post-translational covalent modification of substrates with the small and conserved ubiquitin (Ub), is crucial for the regulation of signaling cascades in numerous cellular processes, including innate immunity and inflammation. Ub conjugation occurs through the combined action of E1 and E2 enzymes and specific E3 Ub ligases, capable of assembling poly-Ub chains linked through eight different Ub linkages, i.e., Lys6, Lys11, Lys27, Lys29, Lys33, Lys48, Lys63, and Met1-linked (linear) Ub. These chains are recognized by proteins via their ubiquitin-binding domains (UBDs) [1] and the position, linkage type and conformation of poly-Ub chains determine their function, ranging from proteasomal degradation to the control of protein-protein interactions, protein activity and signaling [2].

Met1-linked non-degradative ubiquitination is mediated by the linear ubiquitin chain assembly complex (LUBAC). LUBAC and Met1 Ub are closely associated with innate immune receptor signaling and control pro-inflammatory signaling through nuclear factor-κB (NF-κB) or mitogen-activated protein kinases (MAPK), and cell death. Hence, a tight regulation of Met1-linked poly-Ub at receptor signaling complexes is critical for cell fate control and tissue homeostasis, and deregulation of the Met1 Ub machinery is associated with severe inflammatory diseases [3–9].

Ubiquitination is counteracted by deubiquitinating enzymes (DUBs), specialized proteases which remove Ub from target proteins. To date, seven distinct DUB families have been described in humans, i.e., the ubiquitin-specific proteases (USPs), the ovarian tumor (OTU) proteases, the Machado-Joseph Disease (MJD) DUBs, the ubiquitin C-terminal hydrolases (UCHs), the motif interacting with ubiquitin (MIU)-containing novel DUB family (MINDY), the JAB1/MPN/MOV34 metalloprotease DUBs (JAMMs), and the recently discovered zinc finger-containing ubiquitin peptidase 1 (ZUP1) [10]. Most of the known human DUBs show no or only poor activity towards Met1-linked Ub [11–13], with the exception of the USP DUB cylindromatosis (CYLD), which cleaves Lys63 and Met1-linked poly-Ub chains with similar activity, and OTULIN (OTU DUB with linear specificity, also called FAM105B or Gumby) [14–17]. OTULIN is, up till now, the only mammalian DUB described to exclusively hydrolyze Met1-linked poly-Ub chains, thereby counteracting the generation of Met1-linked Ub by LUBAC [16, 17]. OTULIN directly interacts with LUBAC and regulates immune receptor signaling. Accordingly, OTULIN deficiency results in severe auto-inflammatory disease in patients with OTULIN-related auto-inflammatory syndrome (ORAS, also termed otulipenia) and in mouse models [7–9, 18–20]. Intriguingly, new roles for OTULIN have been described in the control of type I interferon (IFN) signaling and antiviral responses [21–23], autophagy [24] and mammalian target of rapamycin (mTOR) signaling [25]. Furthermore, OTULIN activity is not only restricted to Ub-dependent functions. OTULIN has been implicated to regulate cellular trafficking [26] and the pseudo-DUB OTULINL (FAM105A) localizes to the endoplasmic reticulum (ER), where it supposedly mediates protein-protein interactions [27]. Here, we summarize the current knowledge of OTULIN-mediated cell fate control and discuss novel regulatory mechanisms and emerging Ub-dependent and -independent functions of OTULIN.

## Structure-function relationships of OTULIN

**Fig. 1:**
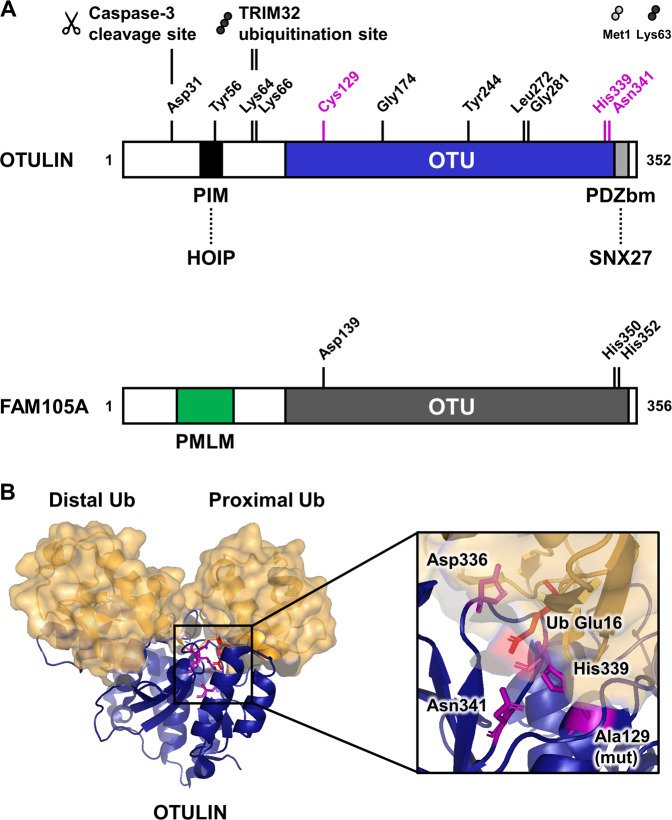
Structural and mechanistic characteristics of OTULIN/Gumby/FAM105B and the OTULIN-related pseudo-deubiquitinating enzyme OTULINL/FAM105A. **a** Schematic comparison of the domain structures, important residues and interaction regions of human OTULIN/Gumby/FAM105B and the pseudo-DUB OTULINL/FAM105A. The N-terminus of OTULIN contains the PUB-interacting motif (PIM) that mediates the interaction with the HOIP subunit of LUBAC and includes the Tyr56 phosphorylation site. In addition, OTULIN contains an N-terminal caspase-3 cleavage site at Asp31 and an ubiquitination site at Lys64/66 which is targeted by TRIM32. The C-terminal OTU domain of OTULIN contains a catalytic triad consisting of Cys129/His339/Asn341 and a PDZ binding motif (PDZbm), responsible for binding to SNX27. Mutation of residues Gly174, Tyr244, Leu272, or Gly281 results in catalytically inactive OTULIN or reduced protein stability, causing the severe inflammatory phenotype in ORAS patients. In contrast, the pseudo-DUB FAM105A OTU domain contains a disrupted catalytic triad (Asp139/His350/His352) that lacks catalytic activity towards any di-ubiquitin linkage. FAM105A contains an N-terminally predicted membrane localization motif (PMLM). **b** Structural representation of the OTULIN OTU domain (blue; with Cys129 mutated to Ala) in complex with Met1-linked di-Ub (yellow) (PDB code: 3znz) [16] with a close-up view of the catalytic triad. Substrate specificity is mediated through Ub-assisted catalysis, whereby, in the presence of Met1-linked di-Ub, the Glu16 residue of the proximal Ub displaces the inhibitory residue Asp336 and coordinates the catalytic residue Asn341 to activate the catalytic triad (Cys129/His339/Asn341) of OTULIN.

The 352-amino acid protein OTULIN has been identified by Keusekotten et al. in a bioinformatics approach to identify previously uncharacterized OTU DUBs [16]. In parallel, Rivkin et al. demonstrated that mutations in the OTULIN gene cause the *gumby* phenotype in mice, resulting in embryonic death after embryonic day E12.5 due to defective development of the cranial vasculature [17]. OTULIN contains a highly conserved catalytic OTU domain that shares features of the papain-like family of cysteine peptidases [28] and employs a Cys129/His339/Asn341 catalytic triad [16, 17]. Apart from a PDZ (PSD95–Dlg1–ZO-1) domain-binding motif at the C-terminus [17, 26], OTULIN contains a peptide:N-glycanase (PNGase)/UBA-containing or UBX-containing proteins (PUB) interaction motif (PIM) at the N-terminus, that includes a phosphorylation site at Tyr56 involved in LUBAC binding (see below) [18, 19] (Fig. 1a). Intriguingly, OTULIN exclusively hydrolyzes Met1-linked poly-Ub chains, without affecting other Ub linkage types [11, 16, 17], including the structurally similar Lys63-linked poly-Ub chains, even at high enzyme concentrations [16, 17]. The striking specificity of OTULIN for Met1-linked poly-Ub originates from a highly conserved Ub binding site that discriminates between Met1-linked and Lys63-linked Ub [16]. Upon binding to Ub, the S1’ binding site in OTULIN orientates the Met1 residue of the proximal Ub towards the catalytic center (Fig. 1b), while the inter-Ub Lys residues remain remote from the catalytic center, except for Lys63, which is fixed in a dedicated binding pocket [16]. Binding of Ub chains with different linkages, even the structurally similar Lys63-linked chains, would rotate the proximal Ub for several degrees and restrict proper binding to the S1’ binding site [16]. Therefore, the conformation of the Ub binding pocket of OTULIN already contributes significantly to its specificity, which is reflected by the 100-fold reduced affinity of Lys63-linked di-Ub (*K*_D_: 12 µM) compared to Met1-linked di-Ub (*K*_D_: 120 nM) [16]. Apart from regulation by post-translational modifications, like phosphorylation, oxidation, ubiquitination and SUMOylation, DUB activity is also controlled by allosteric effects [29]. In addition, Ub has been demonstrated to directly contribute to DUB activation, and this substrate-assisted catalysis has first been reported for OTULIN [16, 17]. Similar mechanisms have been described for the Lys11-specific OTU DUB Cezanne (OTUD7B) as well [30]. In the absence of Ub, OTULIN Cys129, His339, and Asn341 adopt an inhibited catalytic triad conformation, where non-catalytic Asp336 restricts His339 from its catalytic position and Cys129 is present as an inactive rotamer [16]. This auto-inhibition is released upon binding to Met1-linked proximal Ub and the steric interference by its carbonyl group [16]. More specifically, the side chain of Glu16 of the proximal Met1-linked Ub inserts into the catalytic center of OTULIN to displace the inhibitory Asp336 and to activate the catalytic His339, leading to deprotonation of catalytic Cys129 [16] (Fig. 1b). Moreover, Ub Glu16 coordinates catalytic Asn341, resulting in activation of OTULIN’s catalytic triad [16] (Fig. 1b). Notably, mutation of Ub Glu16 to Ala leads to a 240-fold decrease in the turnover number (k_cat_), confirming the critical role of Ub Glu16 for catalysis [16]. Together, the remarkable specificity of OTULIN for Met1 Ub and the Met1-Ub-assisted catalysis suggest that Met1-linked poly-Ub needs to be tightly regulated in cells [16]. Indeed, extensive research conducted over the past decade confirmed the critical roles of Met1-linked poly-ubiquitination in cell fate control, tissue homeostasis and other cellular processes (see below) [31, 32].

**Fig. 2:**
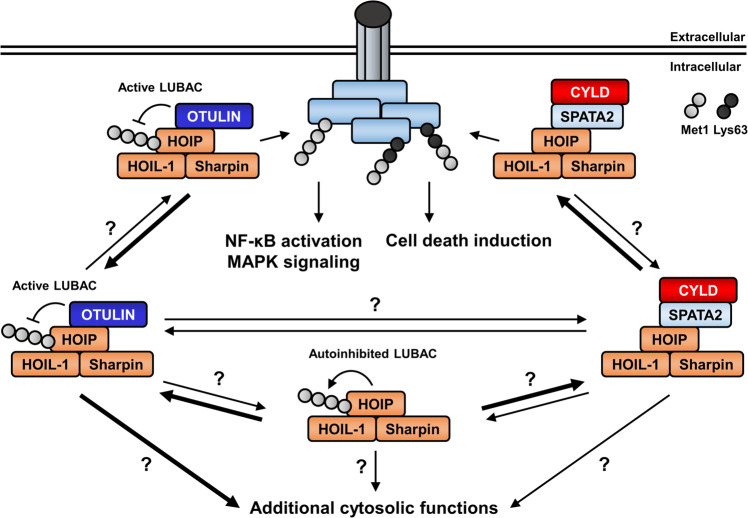
Met1 Ub-processing DUBs are distributed in distinct cellular localization patterns that correspond with their biological function. Mutually exclusive OTULIN- and CYLD-containing LUBAC complexes have been identified in different cellular locations and settings. The biological functions of these complexes depend on the DUB that interacts with LUBAC and range from the control of LUBAC auto-inhibition and activity, as well as modification of membrane- or cytosol-based substrates that control cell fate and additional processes. At present, it remains unclear (indicated by question marks) if OTULIN and CYLD are statically complexed with LUBAC or exist in dynamic equilibriums, regulated by context-type and cell-type specific determinants.

Genes encoding *OTULIN* can be found in organisms of the vertebrate lineage, as well as in a few non-chordates, and these taxa also carry genes encoding the LUBAC components [16], suggesting a potential co-evolution. LUBAC is a multi-protein complex consisting of Sharpin (shank-associated RH domain-interacting protein), HOIL-1 (RBCK1) and HOIP (HOIL-1-interacting protein, RNF31) [5, 6, 33, 34]. Within the complex, HOIP supplies the E3 ligase activity responsible for Met1-linked ubiquitination through a C-terminal really interesting new gene (RING)/in-between RING (IBR)/RING motif [35]. In addition, a conserved linear ubiquitin chain-determining domain (LDD) contributes to the specificity of HOIP for producing Met1-linked poly-Ub chains [36, 37]. HOIP contains a ubiquitin-associated (UBA) domain, several zinc finger domains and an N-terminal PUB domain that mediates binding to OTULIN [35]. OTULIN selectively interacts with LUBAC through the N-terminal PIM, covering Asp54-Met55-Tyr56-Arg57-Ala58 [18, 19], but is not able to interact with the PUB domains of UBX domain-containing protein 1 (UBXD1) or PNGase. Although HOIP is able to interact with the AAA ATPase p97/valosin-containing protein (VCP) [18, 19], which has recently been implicated in the recruitment of LUBAC to misfolded Huntingtin species [38], the HOIP PUB domain binds OTULIN with a 40-fold higher affinity compared to p97/VCP and NMR-based *in vitro* studies indicate a more stable interaction between HOIP and OTULIN compared to p97/VCP [19]. The specificity of the HOIP PUB-OTULIN interaction stems from a binding preference of the HOIP PIM pocket for internal PIMs in contrast to the C-terminal PIM of p97/VCP [18, 19]. The PIM is the sole interaction site between OTULIN and HOIP, and the OTU domain is dispensable for binding of HOIP [16, 17]. Consistently, mutation of the OTULIN catalytic Cys129 does not affect the association with HOIP [18]. Intriguingly, LUBAC also uses PUB-PIM interactions to interact with CYLD through the adapter spermatogenesis-associated protein 2 (SPATA2) [39–41]. Therefore, three different interaction profiles for the HOIP PUB domain have been identified so far, suggesting that there may exist at least three different cellular pools of LUBAC, possibly with different functions depending on cellular context or compartment. Up till now, the underlying mechanisms controlling the abundance and distribution of these distinct complexes remain largely elusive. Importantly, endogenous OTULIN co-immunoprecipitates with HOIP and tumor necrosis factor receptor 1 (TNFR1) after stimulation with tumor necrosis factor α (TNFα) [16, 18] and has been detected in TNFR1 receptor signaling complexes (RSCs) by LC-MS/MS (liquid chromatography mass spectrometry) after pulldown experiments using FLAG-tagged TNFα [42]. In addition, endogenous OTULIN and LUBAC have been shown to be part of the nucleotide-binding and oligomerization domain-containing protein 2 (NOD2) receptor complex in cells overexpressing HA-tagged NOD2 [43]. Accordingly, CYLD is recruited to TNFR1 and NOD2 RSCs in a HOIP-dependent manner, as well, where it targets Lys63- and Met1-linked poly Ub chains [44, 45]. In contrast to these findings, Draber et al. demonstrated that, although both OTULIN and CYLD interact with HOIP under basal conditions, OTULIN is absent from RSCs [44] (Fig. 2) and that knock-out of OTULIN resulted in an increase of Met1-linked poly-Ub chains in the cytosol but not at TNFR1 or NOD2 RSCs [44]. Since both SPATA2 and OTULIN recognize the same binding motif in the PUB of HOIP, interaction of HOIP with SPATA2/CYLD and OTULIN is mutually exclusive [18, 19, 39, 44]. HOIP (120 kDa), HOIL-1 (58 kDa) and Sharpin (40 kDa) form a high-molecular-mass complex of ~600 kDa, indicating that LUBAC may contain more than one HOIP molecule [19, 34, 45]. Given that, LUBAC may form a putative complex which includes both OTULIN and CYLD bound to two HOIP molecules [45, 46]. However, OTULIN’s activity at RSCs is likely dynamic or time-dependent, and the interaction with LUBAC appears to be tightly regulated (Fig. 2).

Intriguingly, the relevance of OTULIN for Met1-linked poly-Ub and control of pro-inflammatory NF-κB signaling largely depends on the interaction with LUBAC. Mutation of Tyr56 in the OTULIN PIM domain abrogates HOIP binding, resulting in reduced ability of OTULIN to inhibit NF-κB activity and reduced cell death signaling [18, 19, 46, 47]. OTULIN, phosphorylated at Tyr56, displays reduced binding to HOIP, leading to HOIP auto-ubiquitination [18, 19]. Notably, neither mutation of Tyr56 nor phosphorylation of OTULIN affects the catalytic activity of OTULIN *in vitro* [18, 47], suggesting that Tyr56 phosphorylation mainly regulates the OTULIN–LUBAC interaction. Interestingly, OTULIN is hyper-phosphorylated at Tyr56 during necroptosis, pointing towards a role of OTULIN Tyr56 phosphorylation in the regulation of necroptotic cell death [47] (see below). Although the responsible kinase(s) have not yet been identified, the dual specificity protein phosphatase 14 (DUSP14) has been reported to dephosphorylate OTULIN [47].

The OTULIN–HOIP interaction is also modulated by ubiquitination of OTULIN by the E3 ligase tripartite motif-containing protein 32 (TRIM32). TRIM32 interacts with the OTULIN OTU domain and conjugates non-proteolytic Lys63-linked poly-Ub at two lysine residues (Lys64 and Lys66) in the vicinity of the PIM domain (Fig. 1a), thereby reducing the HOIP interaction and enhancing TNFα-induced NF-κB activation [48]. Importantly, stimulation with TNFα increased the OTULIN–TRIM32 interaction, indicating that TRIM32-mediated ubiquitination contributes to the control of inflammatory signaling [48].

## Ubiquitin-dependent functions of OTULIN in immune signaling and cell fate control

**Fig. 3:**
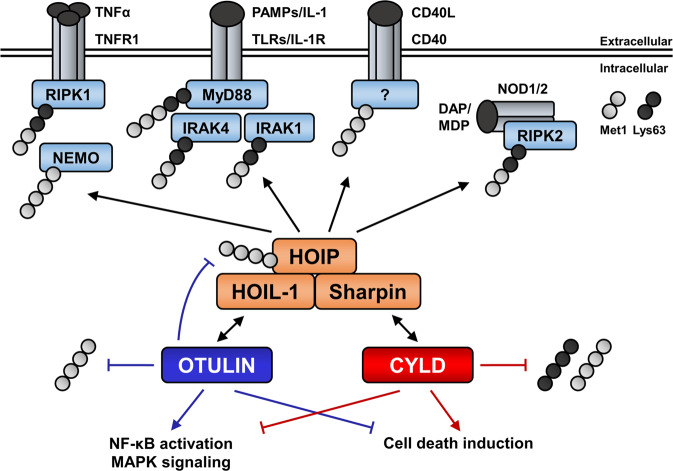
Control of Met1-linked ubiquitin by OTULIN–LUBAC and CYLD-LUBAC complexes regulates innate immune signaling pathways and cell fate. LUBAC is recruited to immune receptors, including tumor necrosis factor receptor 1 (TNFR1), toll-like receptors (TLRs), the interleukin-1 receptor (IL-1R), CD40 and nucleotide-binding and oligomerization domain-containing protein 1/2 (NOD1/2) through HOIP, where it mediates Met1-linked ubiquitination of receptor-associated proteins and pre-existing Lys63 Ub chains. TNFR1 activation leads to recruitment and LUBAC-mediated Met1-linked ubiquitination of RIPK1 and NEMO, whereas upon stimulation of TLRs and IL-1R the adapter protein MyD88 and kinases IRAK1 and IRAK4 are recruited to the respective receptor and targeted by LUBAC. Activation of the cytosolic pattern recognition receptors NOD1/2 results in LUBAC-mediated ubiquitination of RIPK2. Although LUBAC plays important roles at CD40 receptor complexes, the Met1-linked Ub targets remain unclear. Ubiquitination at innate immune receptors is counteracted by the deubiquitinating enzymes OTULIN and CYLD. OTULIN targets Met1-linked Ub and counteracts LUBAC auto-ubiquitination, thereby promoting NF-κB activation and MAPK signaling. In contrast, CYLD targets both Met1-linked and Lys63-linked Ub, leading to destabilization of receptor signaling complexes and thus inhibition of pro-survival signaling via NF-κB and MAPK signaling and induction of cell death signaling.

Engagement of immune receptors, like TNFR1, NOD-like receptors (NLRs), CD40, toll-like receptors (TLRs) and the interleukin-1 receptor (IL-1R), initiates the formation of RSCs in which Lys63 Ub-dependent recruitment of LUBAC is a key event that allows Met1-linked ubiquitination of TNFR1, receptor-associated proteins or existing Lys63 poly-Ub chains [32, 44, 49–51] (Fig. 3). These Lys63 and Met1 Ub chains subsequently recruit the transforming growth factor β (TGFβ)-activated kinase 1 (TAK1) complex and inhibitor of κB kinase (IKK) complex to allow downstream MAPK and NF-κB signaling [49, 50, 52, 53]. The ability of OTULIN to counteract LUBAC-mediated Met1 ubiquitination and NF-κB activation has been demonstrated in numerous studies [7–9, 16–19, 43, 45, 46]. Consistently, OTULIN deficiency or overexpression of catalytically inactive OTULIN mutants strikingly induce cellular Met1 Ub levels [8, 9, 16, 17, 19, 24, 43, 44], as well as accumulation of Met1-linked Ub on several key proteins of inflammatory pathways, including TNFR1, receptor-interacting serine/threonine-protein kinase-1 (RIPK1), receptor-interacting serine/threonine-protein kinase-2 (RIPK2), NF-κB essential modulator (NEMO) and LUBAC [9, 16, 19, 23, 43–45, 47]. However, recent studies revealed that the role of OTULIN in the context of NF-κB activation and cell fate control is more complex and cell type-specific. Initially, OTULIN has been described as a negative regulator of NF-κB-mediated inflammatory responses [16]. TNFα-mediated NF-κB activation and gene transcription decrease upon ectopic expression of OTULIN, while OTULIN knockdown enhances NF-κB activation and LUBAC auto-ubiquitination [16]. In contrast to CYLD and the Ub-modulating enzyme A20 (TNFAIP3), which counteract NF-κB signaling through a negative feedback loop, OTULIN is not transcriptionally regulated by NF-κB [16, 17, 43, 54, 55]. This might suggest that OTULIN primarily controls basal Met1 Ub levels under non-stimulated conditions, but OTULIN has been shown to regulate Met1-linked Ub on RIPK2 upon stimulation of NOD2 [43]. In contrast, although RIPK1 has been described to be targeted by LUBAC [5, 16, 56], RIPK1 Met1 Ub levels are only slightly altered in OTULIN knockdown cells upon TNFα treatment [16]. Interestingly, overexpression of catalytically inactive OTULIN C129A also suppressed NF-κB signaling, in contrast to the Ub-binding mutant OTULIN W96A, likely due to the ability of OTULIN C129A to compete with endogenous UBDs involved in NF-κB signaling, as it is the case for Ub-binding domain in ABIN proteins and NEMO (UBAN)-based Met1 Ub sensors as well [16, 57]. Intriguingly, the notion that OTULIN mainly functions by counteracting LUBAC-mediated activation of pro-inflammatory NF-κB or MAPK signaling has recently been challenged. Heger et al. demonstrated that NF-κB activation upon TNFα treatment is reduced in OTULIN^C129A/C129A^ mouse embryonic fibroblasts, while apoptotic and necroptotic cell death is increased [23], suggesting a destabilization of TNFR complex I. In line with these observations, TNFα stimulation results in reduced Met1 ubiquitination of RIPK1 and NEMO, and decreased NF-κB signaling in OTULIN-deficient keratinocytes [47]. Strikingly, OTULIN deficiency or expression of catalytic inactive OTULIN, but not CYLD deficiency, results in Met1 ubiquitination of LUBAC components [7, 16, 19, 23, 43–45, 47]. This is also the case when OTULIN is phosphorylated at Tyr56 and in cells expressing the binding mutant OTULIN Y56F, both of which disrupt the OTULIN–HOIP interaction [18, 19]. Thus, active OTULIN is required to interact with LUBAC in order to inhibit LUBAC auto-ubiquitination (Fig. 2). Recent studies also indicate that loss of OTULIN expression correlates with reduced abundance of LUBAC components in fibroblasts and in B-cells and T-cells, whereas LUBAC levels in OTULIN-deficient myeloid cells remain stable [7–9, 23]. Concomitant loss of LUBAC in OTULIN-deficient fibroblasts and lymphocytes prevents the hyper-inflammatory phenotype observed in OTULIN-deficient myeloid cells [7, 8]. Together, these findings reveal that OTULIN promotes rather than counteracts LUBAC function by preventing its auto-ubiquitination (Fig. 2). Moreover, these observations also highlight an intricate relationship between OTULIN and LUBAC protein abundance and stability.

Met1-linked Ub abundance at RSCs appears to be decisive for cell fate, as decreased Met1-linked Ub results in destabilization of complex I upon TNFR1 activation and initiation of apoptotic or necroptotic cell death [58]. Consistent with the role of OTULIN in maintaining LUBAC activity, loss of OTULIN or expression of catalytically inactive OTULIN render cells hypersensitive towards TNFα/cycloheximide (CHX)-induced apoptosis and TNFα/CHX/zVAD-induced necroptosis [47]. Surprisingly, overexpression of wild-type OTULIN abrogates the RIPK1-NEMO interaction and sensitizes to TNFα-induced cell death, as well [16]. Intriguingly, several post-translational modifications and events on OTULIN have recently been discovered (Fig. 1a) that affect TNFR1-mediated cell death. For example, OTULIN becomes cleaved at Asp31 by caspase-3 during TNFα/CHX-induced or UVB irradiation-induced apoptosis, resulting in reduced apoptosis and accumulation of the anti-apoptotic protein cellular FLICE (FADD-like IL-1β-converting enzyme)-inhibitory protein (c-FLIP) [47]. c-FLIP itself is a substrate of LUBAC and remains protected from proteasomal degradation by Met1-linked Ub [59]. In addition, OTULIN becomes hyper-phosphorylated at Tyr56 upon induction of necroptosis, whereby it promotes necroptosis, supposedly by regulating RIPK1 Ub levels [47]. Tyr56 phosphorylation is counteracted by DUSP14 [47], a phosphatase which is also involved in the regulation of NF-κB signaling by dephosphorylating TAK1 and TAK1-binding protein 1 (TAB1) [60, 61]. Interestingly, recent findings also suggest that OTULIN deficiency results in spontaneous inhibitory phosphorylation of CYLD, thereby limiting CYLD activity, which can be rescued by LUBAC inhibition [47]. Altogether, these findings provide valuable and novel insights in how OTULIN controls immune receptor signaling in cell-dependent and context-dependent manners. Nevertheless, it still remains unclear if OTULIN is part of RSCs, whether its function is restricted to the regulation of LUBAC activity and Met1 Ub levels in the cytosol or if OTULIN has additional Met1 Ub-dependent functions independent of the interaction with LUBAC (Fig. 2).

Apart from its role in TNFR1-mediated NF-κB activation, OTULIN also controls innate immune signaling downstream of pattern recognition receptors, such as NOD2 and TLRs, and IL-1R. OTULIN negatively regulates muramyl dipeptide (MDP)-induced activation of the cytosolic pattern recognition receptor NOD2 [43, 45]. NOD2 activation induces the formation of RSCs composed of RIPK2, TNFR-associated factor 2 (TRAF2) and X-linked inhibitor of apoptosis protein (XIAP). XIAP promotes Lys63-linked ubiquitination of RIPK2 to recruit LUBAC which deposits Met1-linked poly-Ub onto RIPK2 [62] (Fig. 3). Loss of OTULIN leads to Met1-linked poly-Ub of LUBAC and RIPK2, thereby recruiting NEMO to allow increased MDP-induced NF-κB signaling [43, 45]. LUBAC-mediated Met1-linked poly-ubiquitination also plays important roles in lipopolysaccharide (LPS)-mediated TLR and IL-1R signaling [32]. Upon stimulation with IL-1β, IL-1R-associated kinases 1 and 4 (IRAK1/4) and the adapter protein myeloid differentiation primary response gene 88 (MyD88) are modified with Met1 Ub [50] (Fig. 3). Loss of OTULIN expression induces increased cytokine production upon treatment with LPS or staphylococcal enterotoxin B in *ex vivo* experiments [9]. LUBAC also targets the adapter protein apoptosis-associated speck-like protein containing a CARD (ASC) [63]. Met1-linked ubiquitination of ASC is required for nucleotide-binding domain and leucine-rich repeat protein (NLRP) 3 inflammasome formation, which mediates activation of pro-caspase-1, resulting in processing of pro-inflammatory cytokines or induction of pyroptosis [63, 64]. Notably, UVB irradiation of OTULIN-deficient keratinocytes, which induces activation of the NLRP1-caspase-1 inflammasome [65], leads to enhanced caspase-1 processing compared to control cells [47]. In line with this, accumulation of Met1-linked Ub on ASC upon IL-1β treatment in primary cells of ORAS patients, deficient in functional OTULIN (see below), results in increased ASC-mediated inflammation [9]. Therefore, OTULIN appears to play important roles in the control of inflammatory responses, through TLR/IL-1R signaling and inflammasomes, that underlie OTULIN-related inflammatory disorders. However, the exact function of OTULIN in the context of IL-1R and TLR signaling has yet to be determined.

Mice carrying the *OTULIN* loss-of-function mutations W96R and D336E die embryonically after embryonic day E12.5 due to defective Wnt signaling, resulting in an abnormal cranial vasculature [17]. Deficiency in functional OTULIN caused by these mutations increases Met1 Ub levels, suggesting a potential role for the LUBAC/OTULIN axis in canonical Wnt signaling [17, 46]. In addition, OTULIN interacts with disheveled 2 (DVL2), a signal transducer of canonical and non-canonical Wnt receptor signaling [66], via its N-terminal region [17]; but how exactly OTULIN affects Wnt signaling has yet to be determined. Interestingly, OTULIN^C129A/C129A^ mice die at embryonic day E10.5 and show extensive cell death in cells of the yolk sac and placenta [23], and tamoxifen-induced adult CreERT2-*Otulin*^*Lac*Z/flox^ mice become moribund within a day [8], suggesting that the lethality in OTULIN-deficient mice is not restricted to defects in developmental Wnt signaling and may involve extensive cell death as well [23].

The pathophysiological relevance of inflammatory roles of OTULIN is most evident in ORAS. ORAS is a fatal inflammatory disease characterized by episodes of sterile systemic inflammation with recurrent fevers and panniculitis [7–9, 67]. ORAS patients carry *OTULIN* mutations which affect the catalytic activity and protein stability and thus result in disturbance of immune receptor signaling. So far, four *OTULIN* mutations have been identified to cause the ORAS phenotype in humans, Tyr244Cys, Leu272Pro, Gly281Arg, and Gly174Aspfs*2, the latter causes a premature stop codon [7–9] (Fig. 1a). ORAS is a TNFα-driven disease defined by enhanced Met1 Ub levels and a strong inflammatory phenotype in myeloid cells with enhanced NF-κB and MAPK activation, increased cytokine secretion and the presence of auto-antibodies in the serum, indicating autoimmunity [7–9]. Intriguingly, the functional consequences of OTULIN deficiency in ORAS are cell type-specific, as has been demonstrated in both patient-derived cells and mouse models [7, 8]. OTULIN deficiency in fibroblasts, and in B-cells and T-cells results in proteasomal degradation of LUBAC components [7–9]. Accordingly, these cell types lack a strong inflammatory phenotype, suggesting that LUBAC destabilization prevents excessive Met1 Ub-dependent signaling [7–9]. Furthermore, cell death may contribute to the ORAS pathophysiology as well, since apoptotic cell death could be detected in the skin of an ORAS patient during ORAS-dependent inflammation and patient-derived OTULIN-deficient fibroblasts are sensitized to TNFα/CHX-induced apoptosis [7]. Consistently, ORAS can be successfully treated with TNFα-blocking drugs [7–9].

## Emerging and novel functions of OTULIN

**Fig. 4:**
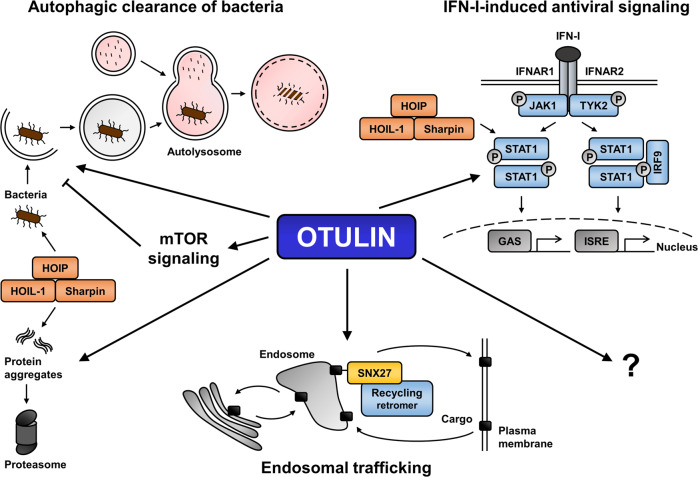
Emerging and novel functions of OTULIN. Recent data link OTULIN-mediated regulation of Met1 Ub to the control of autophagic clearance of bacteria (xenophagy) and degradation of protein aggregates. In addition, OTULIN has been implicated in the regulation of mTOR signaling in the context of liver pathologies. Furthermore, OTULIN-mediated control of Met1 ubiquitination of STAT1 has been shown to regulate IFN-mediated antiviral signaling. Finally, OTULIN interacts with SNX27 to regulate endosome-to-plasma membrane trafficking, thus revealing a novel Ub-independent cytosolic function of OTULIN.

The severe inflammatory phenotypes in ORAS patients are mainly attributed to deregulation of immune receptor signaling in myeloid cells, leading to TNFα-driven inflammation. Surprisingly, OTULIN deficiency in non-hematopoietic cells resulted in severe liver pathology in mice [25]. Hepatocyte-specific OTULIN knock-out mice develop chronic inflammatory liver disease with premalignant alterations, ultimately leading to hepatocellular carcinoma [25, 68]. Loss of OTULIN expression in mouse hepatocytes increases cellular Met1 Ub levels, decreases LUBAC levels and does not induce NF-κB hyper-signaling, but sensitizes to apoptosis [25, 68], in line with previous reports [7, 23]. Importantly, co-deletion of TNFR1 in OTULIN-deficient mice does not ameliorate liver pathology, suggesting TNFα-independent mechanisms [25, 68]. This is in contrast with dysregulated TNFR1 signaling commonly observed in liver disease and cancer and the fact that liver-specific deletion of CYLD induces TNFR1-mediated liver inflammation and development of hepatocellular carcinoma [69, 70]. Intriguingly, hepatocyte-specific deletion of OTULIN in mice results in aberrant activation of the mTOR pathway (Fig. 4), characterized by phosphorylation of mTOR and mTOR substrates, as well as reduced levels of negative regulators of mTOR [25]. Liver pathology in OTULIN knock-out mice can be rescued by treatment with the mTOR inhibitor rapamycin, confirming important roles for OTULIN in mTOR signaling in the liver [25].

Although the exact mechanisms of how OTULIN controls mTOR signaling remain to be established, there are  clues indicating additional roles of OTULIN and Met1 Ub in regulating autophagy (Fig. 4). For example, Met1-linked ubiquitination has been identified on cytosolic *Salmonella* that escape the *Salmonella*-containing vacuole (SCV) or that come in contact with the host cytosol upon leakage of these SCVs [57]. Although the nature of Met1 Ub substrates around intracellular bacteria remains to be established, just like a potential role of Met1-Lys63 hybrid chains, Met1 poly-Ub around bacteria recruit autophagy receptor proteins, like p62 and optineurin, that mediate autophagic clearance by lysosomal degradation (xenophagy) [71–74]. Importantly, Met1 Ub at the bacterial surface also recruits NEMO to locally activate IKK and NF-ĸB in a manner comparable to death receptor activation [24, 74]. Met1 Ub at bacteria is controlled by LUBAC and OTULIN, clearly demonstrating that the Met1 Ub signaling machinery has important functions in the cytosol outside RSC pathways [24, 74]. Accordingly, it has been demonstrated in a recent study that LUBAC and OTULIN also play important pro-survival roles in the recognition and processing of misfolded proteins associated with neurodegenerative diseases, such as mutated Huntingtin, superoxide dismutase 1 (SOD1) and TAR DNA-binding protein 43 (TDP-43) [38]. Aggregated forms of polyQ-expanded Huntingtin (Htt-polyQ) recruit LUBAC through PUB-mediated interaction with p97/VCP, leading to the deposition of poly Met1 Ub chains on these aggregates [38]. Modification of the aggregate surface with Ub chains repels the transcription factor Sp1 that transcriptionally controls LUBAC subunit expression [38]. Increased Met1 Ub of Htt-polyQ induces proteasomal degradation of aggregates [38]. Importantly, loss of OTULIN expression exerts  cytoprotective functions  by reducing cleaved caspase-3 levels [38]. Although NEMO and optineurin are recruited to Met1-modified Htt-polyQ clusters as well, Met1 ubiquitination of protein aggregates mainly serves proteasomal degradation [38].

Recently, OTULIN has also been described to interact with sorting nexin 27 (SNX27), involved in endosomal protein recycling from the cell surface [26] (Fig. 4). OTULIN binding prevented SNX27 cargo loading, the complexation of SNX27 with the retromer component VPS26A and trafficking of glucose transporter 1 (GLUT1) or the trafficking reporter GFP-SLC1A4 from endosomes to the membrane [26]. Interestingly, SNX27 binding did not affect the catalytic activity of OTULIN, the LUBAC interaction or cytosolic Met1 Ub levels, suggesting novel, catalytically-independent roles of OTULIN in protein recycling and trafficking [26]. Intriguingly, the OTULIN pseudo-deubiquitinating enzyme FAM105A (OTULINL) shares ~33% amino acid sequence identity with OTULIN (Fig. 1a), but lacks Met1 Ub catalytic activity or the ability to interact with Ub or LUBAC [27, 75]. In contrast, FAM105A employs a predicted membrane localization motif (PMLM) that directs the protein to membranes [27]. Indeed, FAM105A locates to the cytosol, the nuclear envelope and ER-like membrane and interacts with proteins involved in mediating ER-organelle contacts [27]. At present, it remains unclear why an OTULIN-like pseudo-DUB is expressed in cells and might locate to cellular membranes and further research is required to uncover these functions.

The cytosolic roles of OTULIN and Met1 Ub are not only restricted to control “non-self” responses to bacteria or protein aggregates or protein trafficking, but recent findings also link the linear Ub machinery to antiviral signaling and expression of antiviral cytokines, including type-I IFNs (Fig. 4). LUBAC and Met1-linked Ub are implicated in virus-induced retinoic acid-inducible gene I (RIG-I)-dependent IFN production [76, 77]. Virus infection can induce the upregulation of HOIP to enhance HOIP-mediated Met1-linked ubiquitination of signal transducer and activator of transcription 1 (STAT1) [21]. Ubiquitination of STAT1 blocks the STAT1-IFNα/β receptor 2 (IFNAR2) interaction and IFN-I-induced antiviral activity [21]. Importantly, OTULIN overexpression results in decreased Met1 Ub levels of STAT1, leading to enhanced STAT1-IFNAR2 interaction and expression of IFN-stimulated genes upon IFN-I treatment [21]. Consistently, OTULIN knockdown blocks STAT1-IFNAR2 interaction and IFN-I-mediated STAT1 activation, demonstrating that OTULIN regulates STAT1 Met1-linked ubiquitination during IFN-I-induced antiviral signaling [21].

Interestingly, liver inflammation observed in OTULIN-deficient mice is accompanied by increased expression of IFN response genes [68] and murine *Otulin*^*C129A/C129A*^ *Ripk3*^−/−^ *Casp8*^−/−^ BMDMs show spontaneous activation of IKKε and TANK-binding kinase 1 (TBK1) [23], indicating that OTULIN suppresses IFN-I production pathways. Indeed, stimulation of *Otulin*^*C129A/C129A*^ *Ripk3*^−/−^ *Casp8*^−/−^ BMDMs with the RIG-I ligand ppp-dsRNA results in enhanced production of type-I IFN compared to control cells [23].

In contrast, the porcine reproductive and respiratory syndrome virus (PRRSV) hijacks porcine OTULIN to negatively regulate innate immunity during PRRSV infection. OTULIN is upregulated in a PRRSV infection model and interacts with the PRRSV non-structural protein 11 (Nsp11) to decrease Ub levels on NEMO and to block NF-κB activation and IFN-regulatory transcription factor 3 (IRF3)-mediated type-I IFN production [22]. Furthermore, using a genome-wide functional knock-out screen, OTULIN has recently been implicated in the reactivation of HIV-1 in a HIV-1 latency cell line model [78].

## Conclusions

Although, in recent years, it became well established that tight control of Met1-linked ubiquitination serves crucial functions in inflammatory signaling and cell fate, the key player OTULIN remains largely elusive. Cell- and context-dependent differences in catalytic activity, cellular localization, interaction partners, post-translational modifications and cleavage balance the function of OTULIN. Increasing evidence suggests intriguing roles of OTULIN at RSCs, apart from its role in controlling LUBAC activity, but many questions remain unanswered. Since immune receptor signaling networks need to be tightly controlled, LUBAC- and OTULIN-centered interaction networks are dynamic and carefully regulated as well. The spatiotemporal formation of transient protein interactions and complexes, regulated by post-translational modifications, contribute as well to signaling control. It also remains unclear if OTULIN–LUBAC and CYLD–LUBAC are in equilibrium and could exchange DUB subunits or if these are physically different complexes. Loss of OTULIN expression remarkably increases cellular Met1-linked Ub levels, suggesting that OTULIN regulates cytosolic Met1 ubiquitination-dependent processes. Novel Ub-dependent and -independent roles of OTULIN in diverse cellular compartments and organelles are emerging that unexpectedly connect novel cellular and biological functions with a key player of the Met1 Ub machinery.

## References

[1] Husnjak K, Dikic I (2012). Ubiquitin-binding proteins: decoders of ubiquitin-mediated cellular functions. Annu Rev Biochem.

[2] Akutsu M, Dikic I, Bremm A (2016). Ubiquitin chain diversity at a glance. J Cell Sci.

[3] Boisson B, Laplantine E, Dobbs K, Cobat A, Tarantino N, Hazen M (2015). Human HOIP and LUBAC deficiency underlies autoinflammation, immunodeficiency, amylopectinosis, and lymphangiectasia. J Exp Med.

[4] Boisson B, Laplantine E, Prando C, Giliani S, Israelsson E, Xu Z (2012). Immunodeficiency, autoinflammation and amylopectinosis in humans with inherited HOIL-1 and LUBAC deficiency. Nat Immunol.

[5] Gerlach B, Cordier SM, Schmukle AC, Emmerich CH, Rieser E, Haas TL (2011). Linear ubiquitination prevents inflammation and regulates immune signalling. Nature.

[6] Ikeda F, Deribe YL, Skanland SS, Stieglitz B, Grabbe C, Franz-Wachtel M (2011). SHARPIN forms a linear ubiquitin ligase complex regulating NF-kappaB activity and apoptosis. Nature.

[7] Damgaard RB, Elliott PR, Swatek KN, Maher ER, Stepensky P, Elpeleg O (2019). OTULIN deficiency in ORAS causes cell type-specific LUBAC degradation, dysregulated TNF signalling and cell death. EMBO Mol Med.

[8] Damgaard RB, Walker JA, Marco-Casanova P, Morgan NV, Titheradge HL, Elliott PR (2016). The deubiquitinase OTULIN is an essential negative regulator of inflammation and autoimmunity. Cell.

[9] Zhou Q, Yu X, Demirkaya E, Deuitch N, Stone D, Tsai WL (2016). Biallelic hypomorphic mutations in a linear deubiquitinase define otulipenia, an early-onset autoinflammatory disease. Proc Natl Acad Sci USA.

[10] Clague MJ, Urbe S, Komander D (2019). Breaking the chains: deubiquitylating enzyme specificity begets function. Nat Rev Mol Cell Biol.

[11] Ritorto MS, Ewan R, Perez-Oliva AB, Knebel A, Buhrlage SJ, Wightman M (2014). Screening of DUB activity and specificity by MALDI-TOF mass spectrometry. Nat Commun.

[12] Mevissen TE, Hospenthal MK, Geurink PP, Elliott PR, Akutsu M, Arnaudo N (2013). OTU deubiquitinases reveal mechanisms of linkage specificity and enable ubiquitin chain restriction analysis. Cell.

[13] Faesen AC, Luna-Vargas MP, Geurink PP, Clerici M, Merkx R, van Dijk WJ (2011). The differential modulation of USP activity by internal regulatory domains, interactors and eight ubiquitin chain types. Chem Biol.

[14] Komander D, Reyes-Turcu F, Licchesi JD, Odenwaelder P, Wilkinson KD, Barford D (2009). Molecular discrimination of structurally equivalent Lys 63-linked and linear polyubiquitin chains. EMBO Rep.

[15] Sato Y, Goto E, Shibata Y, Kubota Y, Yamagata A, Goto-Ito S (2015). Structures of CYLD USP with Met1- or Lys63-linked diubiquitin reveal mechanisms for dual specificity. Nat Struct Mol Biol.

[16] Keusekotten K, Elliott PR, Glockner L, Fiil BK, Damgaard RB, Kulathu Y (2013). OTULIN antagonizes LUBAC signaling by specifically hydrolyzing Met1-linked polyubiquitin. Cell.

[17] Rivkin E, Almeida SM, Ceccarelli DF, Juang YC, MacLean TA, Srikumar T (2013). The linear ubiquitin-specific deubiquitinase gumby regulates angiogenesis. Nature.

[18] Schaeffer V, Akutsu M, Olma MH, Gomes LC, Kawasaki M, Dikic I (2014). Binding of OTULIN to the PUB domain of HOIP controls NF-kappaB signaling. Mol Cell.

[19] Elliott PR, Nielsen SV, Marco-Casanova P, Fiil BK, Keusekotten K, Mailand N (2014). Molecular basis and regulation of OTULIN-LUBAC interaction. Mol Cell.

[20] Elliott PR, Komander D (2016). Regulation of Met1-linked polyubiquitin signalling by the deubiquitinase OTULIN. FEBS J.

[21] Zuo Y, Feng Q, Jin L, Huang F, Miao Y, Liu J (2020). Regulation of the linear ubiquitination of STAT1 controls antiviral interferon signaling. Nat Commun.

[22] Su Y, Shi P, Zhang L, Lu D, Zhao C, Li R (2018). The superimposed deubiquitination effect of OTULIN and Porcine Reproductive and Respiratory Syndrome Virus (PRRSV) Nsp11 promotes multiplication of PRRSV. J Virol.

[23] Heger K, Wickliffe KE, Ndoja A, Zhang J, Murthy A, Dugger DL (2018). OTULIN limits cell death and inflammation by deubiquitinating LUBAC. Nature.

[24] van Wijk SJL, Fricke F, Herhaus L, Gupta J, Hotte K, Pampaloni F (2017). Linear ubiquitination of cytosolic Salmonella Typhimurium activates NF-kappaB and restricts bacterial proliferation. Nat Microbiol.

[25] Damgaard RB, Jolin HE, Allison MED, Davies SE, Titheradge HL, McKenzie ANJ (2020). OTULIN protects the liver against cell death, inflammation, fibrosis, and cancer. Cell Death Differ.

[26] Stangl A, Elliott PR, Pinto-Fernandez A, Bonham S, Harrison L, Schaub A (2019). Regulation of the endosomal SNX27-retromer by OTULIN. Nat Commun.

[27] Ceccarelli DF, Ivantsiv S, Mullin AA, Coyaud E, Manczyk N, Maisonneuve P (2019). FAM105A/OTULINL is a pseudodeubiquitinase of the OTU-class that localizes to the ER membrane. Structure.

[28] Rawlings ND, Waller M, Barrett AJ, Bateman A (2014). MEROPS: the database of proteolytic enzymes, their substrates and inhibitors. Nucleic Acids Res.

[29] Mevissen TET, Komander D (2017). Mechanisms of deubiquitinase specificity and regulation. Annu Rev Biochem.

[30] Mevissen TET, Kulathu Y, Mulder MPC, Geurink PP, Maslen SL, Gersch M (2016). Molecular basis of Lys11-polyubiquitin specificity in the deubiquitinase Cezanne. Nature.

[31] Rittinger K, Ikeda F (2017). Linear ubiquitin chains: enzymes, mechanisms and biology. Open Biol.

[32] Hrdinka M, Gyrd-Hansen M (2017). The Met1-linked ubiquitin machinery: emerging themes of (de)regulation. Mol Cell.

[33] Tokunaga F, Nakagawa T, Nakahara M, Saeki Y, Taniguchi M, Sakata S (2011). SHARPIN is a component of the NF-kappaB-activating linear ubiquitin chain assembly complex. Nature.

[34] Kirisako T, Kamei K, Murata S, Kato M, Fukumoto H, Kanie M (2006). A ubiquitin ligase complex assembles linear polyubiquitin chains. EMBO J.

[35] Oikawa D, Sato Y, Ito H, Tokunaga F (2020). Linear UBiquitin Code: Its Writer, Erasers, Decoders, Inhibitors, and Implications in Disorders. Int J Mol Sci.

[36] Smit JJ, Monteferrario D, Noordermeer SM, van Dijk WJ, van der Reijden BA, Sixma TK (2012). The E3 ligase HOIP specifies linear ubiquitin chain assembly through its RING-IBR-RING domain and the unique LDD extension. EMBO J.

[37] Stieglitz B, Rana RR, Koliopoulos MG, Morris-Davies AC, Schaeffer V, Christodoulou E (2013). Structural basis for ligase-specific conjugation of linear ubiquitin chains by HOIP. Nature.

[38] van Well EM, Bader V, Patra M, Sanchez-Vicente A, Meschede J, Furthmann N (2019). A protein quality control pathway regulated by linear ubiquitination. EMBO J.

[39] Elliott PR, Leske D, Hrdinka M, Bagola K, Fiil BK, McLaughlin SH (2016). SPATA2 links CYLD to LUBAC, activates CYLD, and controls LUBAC signaling. Mol Cell.

[40] Kupka S, De Miguel D, Draber P, Martino L, Surinova S, Rittinger K (2016). SPATA2-mediated binding of CYLD to HOIP enables CYLD recruitment to signaling complexes. Cell Rep..

[41] Schlicher L, Wissler M, Preiss F, Brauns-Schubert P, Jakob C, Dumit V (2016). SPATA2 promotes CYLD activity and regulates TNF-induced NF-kappaB signaling and cell death. EMBO Rep.

[42] Wagner SA, Satpathy S, Beli P, Choudhary C (2016). SPATA2 links CYLD to the TNF-alpha receptor signaling complex and modulates the receptor signaling outcomes. EMBO J.

[43] Fiil BK, Damgaard RB, Wagner SA, Keusekotten K, Fritsch M, Bekker-Jensen S (2013). OTULIN restricts Met1-linked ubiquitination to control innate immune signaling. Mol Cell.

[44] Draber P, Kupka S, Reichert M, Draberova H, Lafont E, de Miguel D (2015). LUBAC-recruited CYLD and A20 regulate gene activation and cell death by exerting opposing effects on linear ubiquitin in signaling complexes. Cell Rep.

[45] Hrdinka M, Fiil BK, Zucca M, Leske D, Bagola K, Yabal M (2016). CYLD limits Lys63- and Met1-linked ubiquitin at receptor complexes to regulate innate immune signaling. Cell Rep.

[46] Takiuchi T, Nakagawa T, Tamiya H, Fujita H, Sasaki Y, Saeki Y (2014). Suppression of LUBAC-mediated linear ubiquitination by a specific interaction between LUBAC and the deubiquitinases CYLD and OTULIN. Genes Cells.

[47] Douglas T, Saleh M (2019). Post-translational modification of OTULIN regulates ubiquitin dynamics and cell death. Cell Rep.

[48] Zhao M, Song K, Hao W, Wang L, Patil G, Li Q (2020). Non-proteolytic ubiquitination of OTULIN regulates NF-kappaB signaling pathway. J Mol Cell Biol.

[49] Emmerich CH, Bakshi S, Kelsall IR, Ortiz-Guerrero J, Shpiro N, Cohen P (2016). Lys63/Met1-hybrid ubiquitin chains are commonly formed during the activation of innate immune signalling. Biochem Biophys Res Commun.

[50] Emmerich CH, Ordureau A, Strickson S, Arthur JS, Pedrioli PG, Komander D (2013). Activation of the canonical IKK complex by K63/M1-linked hybrid ubiquitin chains. Proc Natl Acad Sci USA.

[51] Wertz IE, Newton K, Seshasayee D, Kusam S, Lam C, Zhang J (2015). Phosphorylation and linear ubiquitin direct A20 inhibition of inflammation. Nature.

[52] Rahighi S, Ikeda F, Kawasaki M, Akutsu M, Suzuki N, Kato R (2009). Specific recognition of linear ubiquitin chains by NEMO is important for NF-kappaB activation. Cell.

[53] Kanayama A, Seth RB, Sun L, Ea CK, Hong M, Shaito A (2004). TAB2 and TAB3 activate the NF-kappaB pathway through binding to polyubiquitin chains. Mol Cell.

[54] Lee EG, Boone DL, Chai S, Libby SL, Chien M, Lodolce JP (2000). Failure to regulate TNF-induced NF-kappaB and cell death responses in A20-deficient mice. Science.

[55] Jono H, Lim JH, Chen LF, Xu H, Trompouki E, Pan ZK (2004). NF-kappaB is essential for induction of CYLD, the negative regulator of NF-kappaB: evidence for a novel inducible autoregulatory feedback pathway. J Biol Chem.

[56] de Almagro MC, Goncharov T, Newton K, Vucic D (2015). Cellular IAP proteins and LUBAC differentially regulate necrosome-associated RIP1 ubiquitination. Cell Death Dis.

[57] van Wijk SJ, Fiskin E, Putyrski M, Pampaloni F, Hou J, Wild P (2012). Fluorescence-based sensors to monitor localization and functions of linear and K63-linked ubiquitin chains in cells. Mol Cell.

[58] Kupka S, Reichert M, Draber P, Walczak H (2016). Formation and removal of poly-ubiquitin chains in the regulation of tumor necrosis factor-induced gene activation and cell death. FEBS J.

[59] Tang Y, Joo D, Liu G, Tu H, You J, Jin J (2018). Linear ubiquitination of cFLIP induced by LUBAC contributes to TNFalpha-induced apoptosis. J Biol Chem.

[60] Zheng H, Li Q, Chen R, Zhang J, Ran Y, He X (2013). The dual-specificity phosphatase DUSP14 negatively regulates tumor necrosis factor- and interleukin-1-induced nuclear factor-kappaB activation by dephosphorylating the protein kinase TAK1. J Biol Chem.

[61] Yang CY, Li JP, Chiu LL, Lan JL, Chen DY, Chuang HC (2014). Dual-specificity phosphatase 14 (DUSP14/MKP6) negatively regulates TCR signaling by inhibiting TAB1 activation. J Immunol.

[62] Damgaard RB, Nachbur U, Yabal M, Wong WW, Fiil BK, Kastirr M (2012). The ubiquitin ligase XIAP recruits LUBAC for NOD2 signaling in inflammation and innate immunity. Mol Cell.

[63] Rodgers MA, Bowman JW, Fujita H, Orazio N, Shi M, Liang Q (2014). The linear ubiquitin assembly complex (LUBAC) is essential for NLRP3 inflammasome activation. J Exp Med.

[64] Guo H, Callaway JB, Ting JP (2015). Inflammasomes: mechanism of action, role in disease, and therapeutics. Nat Med.

[65] Feldmeyer L, Keller M, Niklaus G, Hohl D, Werner S, Beer HD (2007). The inflammasome mediates UVB-induced activation and secretion of interleukin-1beta by keratinocytes. Curr Biol.

[66] Nielsen CP, Jernigan KK, Diggins NL, Webb DJ, MacGurn JA (2019). USP9X deubiquitylates DVL2 to regulate WNT pathway specification. Cell Rep.

[67] Nabavi M, Shahrooei M, Rokni-Zadeh H, Vrancken J, Changi-Ashtiani M, Darabi K (2019). Auto-inflammation in a patient with a novel homozygous OTULIN mutation. J Clin Immunol.

[68] Verboom L, Martens A, Priem D, Hoste E, Sze M, Vikkula H (2020). OTULIN prevents liver inflammation and hepatocellular carcinoma by inhibiting FADD- and RIPK1 kinase-mediated hepatocyte apoptosis. Cell Rep.

[69] Nikolaou K, Tsagaratou A, Eftychi C, Kollias G, Mosialos G, Talianidis I (2012). Inactivation of the deubiquitinase CYLD in hepatocytes causes apoptosis, inflammation, fibrosis, and cancer. Cancer Cell.

[70] Luedde T, Kaplowitz N, Schwabe RF (2014). Cell death and cell death responses in liver disease: mechanisms and clinical relevance. Gastroenterology.

[71] Wild P, Farhan H, McEwan DG, Wagner S, Rogov VV, Brady NR (2011). Phosphorylation of the autophagy receptor optineurin restricts Salmonella growth. Science.

[72] Mostowy S, Sancho-Shimizu V, Hamon MA, Simeone R, Brosch R, Johansen T (2011). p62 and NDP52 proteins target intracytosolic Shigella and Listeria to different autophagy pathways. J Biol Chem.

[73] Zheng YT, Shahnazari S, Brech A, Lamark T, Johansen T, Brumell JH (2009). The adaptor protein p62/SQSTM1 targets invading bacteria to the autophagy pathway. J Immunol.

[74] Noad J, von der Malsburg A, Pathe C, Michel MA, Komander D, Randow F (2017). LUBAC-synthesized linear ubiquitin chains restrict cytosol-invading bacteria by activating autophagy and NF-kappaB. Nat Microbiol.

[75] Walden M, Masandi SK, Pawlowski K, Zeqiraj E (2018). Pseudo-DUBs as allosteric activators and molecular scaffolds of protein complexes. Biochem Soc Trans.

[76] Belgnaoui SM, Paz S, Samuel S, Goulet ML, Sun Q, Kikkert M (2012). Linear ubiquitination of NEMO negatively regulates the interferon antiviral response through disruption of the MAVS-TRAF3 complex. Cell Host Microbe.

[77] Inn KS, Gack MU, Tokunaga F, Shi M, Wong LY, Iwai K (2011). Linear ubiquitin assembly complex negatively regulates RIG-I- and TRIM25-mediated type I interferon induction. Mol Cell.

[78] Rathore A, Iketani S, Wang P, Jia M, Sahi V, Ho DD (2020). CRISPR-based gene knockout screens reveal deubiquitinases involved in HIV-1 latency in two Jurkat cell models. Sci Rep.

